# Supplementary material for: Muscle strength is associated with COVID‐19 hospitalization in adults 50 years of age or older
Source: J Cachexia Sarcopenia Muscle. 2021 Aug 6;12(5):1136–43. doi: 10.1002/jcsm.12738 (PMC8426913; doi:10.1002/jcsm.12738)
Supplement: Supplementary file 1 — Table S1. Cumulative proportion of COVID‐19 cases. Table S2. Results based on average muscle strength (2004–2017). Table S3. Results based on muscle strength assessed in wave 7. Table S4. Results adjusted for the country of residence. Table S5. Results based on the rare events logistic regression with a tau of 84/100,00. Table S6. Results in COVID‐19 patients (N = 289). [file JCSM-12-1136-s001.docx]

**Supplemental Material**

**Table S1.** Cumulative proportion of COVID-19 cases

**Sensitivity analyses**

**Table S2.** Results based on average muscle strength (2004-2017)

**Table S3.** Results based on muscle strength assessed in wave 7

**Table S4.** Results adjusted for the country of residence

**Robustness analyses**

**Table S5.** Results based on the rare events logistic regression with a tau of 84/100,000

**Table S6.** Results in COVID-19 patients (N = 289)

**Table S1.** Cumulative proportion of COVID-19 cases

| **Country** | **Date^*^** | **Cumulative cases^a^** | **Cumulative hospitalizations** | **Country population size in 2020** (rounded in millions)^b^ | **Proportion** (cumulative case / pop size) | **Proportion** (cumulative hospitalization / pop size) |
| --- | --- | --- | --- | --- | --- | --- |
| Belgium | 15 July | 65368 | 19663 | 11.6 | 0.564% | 0.169% |
| Bulgaria | 21 July | 9348 | NA | 6.9 | 0.135% | NA |
| Croatia^**^ | 15 July | 4356 | 601 | 4.1 | 0.106% | 0.015% |
| Cyprus | 15 July | 1046 | 179 | 1.2 | 0.087% | 0.015% |
| Czech Republic | 8 July | 12898 | 4361 | 10.7 | 0.121% | 0.041% |
| Denmark | 10 July | 13189 | 2646 | 5.8 | 0.227% | 0.046% |
| Estonia | 8 July | 2036 | 435 | 1.3 | 0.157% | 0.033% |
| Finland | 10 July | 7342 | NA | 5.5 | 0.133% | NA |
| France | 7 July | 197224 | 95726 | 65.3 | 0.302% | 0.147% |
| Germany | 15 July | 202132 | 23645 | 83.8 | 0.241% | 0.028% |
| Greece | 12 July | 3810 | NA | 10.4 | 0.037% | NA |
| Hungary | 15 July | 4426 | NA | 9.7 | 0.046% | NA |
| Israel | 4 July | 29870 | 4263 | 8.7 | 0.343% | 0.049% |
| Italy | 5 July | 246845 | 88123 | 60.5 | 0.408% | 0.146% |
| Latvia | 15 July | 1215 | 191 | 1.9 | 0.064% | 0.010% |
| Lithuania | 7 July | 2184 | 454 | 2.7 | 0.081% | 0.017% |
| Luxembourg | 15 July | 4493 | NA | 0.6 | 0.749% | NA |
| Malta | 15 July | 633 | 47 | 0.4 | 0.158% | 0.012% |
| Netherlands | 10 July | 51481 | NA | 17.1 | 0.301% | NA |
| Poland | 8 July | 37054 | NA | 37.8 | 0.098% | NA |
| Portugal | 11 July | 46234 | 4809 | 10.2 | 0.453% | 0.047% |
| Romania | 9 July | 31223 | 17023 | 19.2 | 0.163% | 0.089% |
| Slovakia | 7 July | 1816 | NA | 5.5 | 0.033% | NA |
| Slovenia | 9 July | 1782 | 379 | 2.1 | 0.085% | 0.018% |
| Spain | 10 July | 257675 | 110718 | 46.8 | 0.551% | 0.237% |
| Sweden | 15 July | 77909 | NA | 10.1 | 0.771% | NA |
| Switzerland | 15 July | 33913 | 4104 | 8.7 | 0.390% | 0.047% |
|  | Total: | 1347502 | 377367 | 448.6 | 0.300% | 0.084% |

*Note.* ^a^“COVID-19 Daily Epidemic Forecasting”, by the [Institute of Global Health](https://www.unige.ch/medecine/isg/en/), of the University of Geneva and the [Swiss Data Science Center](https://datascience.ch/), ETH Zürich-EPFL. ^b^United Nations Population Fund ^c^ Our World in Data (based on weekly numbers). ^*^The date was selected to fall around the middle of the data collection period of the country; ^**^Hospitalization data started in May 2020 (no hospitalization data were available before this period).

**Table S2.** Results based on average muscle strength (2004-2017)

|  | Model 1  (without muscle strength) | | Model 2  (with muscle strength) | |
| --- | --- | --- | --- | --- |
| **Variables** | OR (95% CI) | *p*-value | OR (95% CI) | *p*-value |
| Intercept | .015 (.01; .02) | <.001 | .012 (.01; .02) | <.001 |
| Age | 1.70 (1.32; 2.20) | <.001 | 1.50 (1.15; 1.97) | .003 |
| Sex (ref. Female) |  |  |  |  |
| Male | .79 (.43; 1.43) | .427 | 1.35 (.64; 2.79) | .419 |
| Height | 1.31 (.96; 1.77) | .089 | 1.48 (1.07; 2.04) | .016 |
| Body mass index (ref. Normal) |  |  |  |  |
| Overweight | 1.44 (.85; 2.49) | .179 | 1.56 (.92; 2.71) | .104 |
| Obese | 2.01(1.09; 3.69) | .025 | 2.15 (1.16; 3.97) | .014 |
| Cardiovascular disease (ref. No) |  |  |  |  |
| Yes | 1.06 (.66; 1.73) | .811 | 1.02 (.63; 1.67) | .922 |
| Respiratory disease (ref. No) |  |  |  |  |
| Yes | 1.52 (.66; 3.08) | .278 | 1.53 (.66; 3.09) | .278 |
| Diabetes (ref. No) |  |  |  |  |
| Yes | 1.19 (.59; 2.19) | .601 | 1.09 (.55; 2.02) | .787 |
| Cancer (ref. No) |  |  |  |  |
| Yes | .67 (.16; 1.85) | .504 | .62 (.15; 1.73) | .431 |
| Rheumatoid arthritis (ref. No) |  |  |  |  |
| Yes | 1.17 (.55; 2.24) | .669 | 1.10 (.51; 2.11) | .798 |
| Chronic kidney disease (ref. No) |  |  |  |  |
| Yes | 2.44 (.71; 6.41) | .104 | 2.36 (.68; 6.20) | .118 |
| Muscle strength |  |  | .62 (.42; .92) | .017 |

**Table S3.** Results based on muscle strength assessed in wave 7

| **N = 2’884** | Model 1  (without muscle strength) | | Model 2  (with muscle strength) | |
| --- | --- | --- | --- | --- |
| **Variables** | OR (95% CI) | *p*-value | OR (95% CI) | *p*-value |
| Intercept | .016 (.01; .03) | <.001 | .012 (.01; .02) | <.001 |
| Age | 1.50 (1.11; 1.99) | <.007 | 1.31 (.96; 1.78) | .086 |
| Sex (ref. Female) |  |  |  |  |
| Male | .81 (.41; 1.59) | .538 | 1.31 (.58; 2.85) | .507 |
| Height | 1.25 (.87; 1.77) | .222 | 1.38 (.96; 1.97) | .079 |
| Body mass index (ref. Normal) |  |  |  |  |
| Overweight | 1.53 (.84; 2.85) | .169 | 1.62 (.89; 3.03) | .119 |
| Obese | 2.12 (1.07; 4.22) | .031 | 2.24 (1.13; 4.47) | .021 |
| Cardiovascular disease (ref. No) |  |  |  |  |
| Yes | 1.02 (.60; 1.75) | .933 | .98 (.58; 1.69) | .955 |
| Respiratory disease (ref. No) |  |  |  |  |
| Yes | 1.06 (.31; 2.66) | .915 | 1.05 (.31; 2.65) | .920 |
| Diabetes (ref. No) |  |  |  |  |
| Yes | 1.34 (.62; 2.62) | .421 | 1.21 (.56; 2.39) | .589 |
| Cancer (ref. No) |  |  |  |  |
| Yes | .96 (.23; 2.67) | .944 | .90 (.22; 2.53) | .865 |
| Rheumatoid arthritis (ref. No) |  |  |  |  |
| Yes | .99 (.37; 2.18) | .973 | .89 (.33; 1.99) | .801 |
| Chronic kidney disease (ref. No) |  |  |  |  |
| Yes | .87 (.05; 4.22) | .891 | .88 (.05; 4.27) | .899 |
| Muscle strength |  |  | .65 (.44; .96) | .029 |

**Table S4.** Results adjusted for the country of residence

|  | Model 1  (without muscle strength) | | Model 2  (with muscle strength) | |
| --- | --- | --- | --- | --- |
| **Variables** | OR (95% CI) | *p*-value | OR (95% CI) | *p*-value |
| Intercept | .016 (.01; .03) | <.001 | .013 (.01; .02) | <.001 |
| Age | 1.84 (1.41; 2.39) | <.001 | 1.63 (1.23; 2.16) | <.001 |
| Sex (ref. Female) |  |  |  |  |
| Male | .74 (.39; 1.39) | .696 | 1.16 (.55; 2.44) | .696 |
| Height | 1.38 (0.99; 1.92) | .022 | 1.48 (1.06; 2.07) | .022 |
| Body mass index (ref. Normal) |  |  |  |  |
| Overweight | 1.38 (.84; 2.42) | .185 | 1.45 (.84; 2.54) | .185 |
| Obese | 2.00 (1.05; 3.79) | .026 | 2.06 (1.09; 3.91) | .026 |
| Cardiovascular disease (ref. No) |  |  |  |  |
| Yes | 1.02 (.62; 1.69) | .993 | 0.98 (.61; 1.65) | .993 |
| Respiratory disease (ref. No) |  |  |  |  |
| Yes | 1.56 (.65; 3.26) | .284 | 1.55 (.65; 3.24) | .284 |
| Diabetes (ref. No) |  |  |  |  |
| Yes | 1.16 (.57; 2.19) | .782 | 1.10 (.54; 2.08) | .782 |
| Cancer (ref. No) |  |  |  |  |
| Yes | .71 (.17; 2.02) | .490 | .65 (.15; 1.87) | .482 |
| Rheumatoid arthritis (ref. No) |  |  |  |  |
| Yes | 1.21 (.53; 2.47) | .707 | 1.16 (.51; 2.37) | .707 |
| Chronic kidney disease (ref. No) |  |  |  |  |
| Yes | 1.52 (.42; 4.26) | .502 | 1.47 (.41; 4.13) | .502 |
| Muscle strength |  |  | .67 (.46; .98) | .037 |
| Country of residence (ref. Belgium) |  |  |  |  |
| Bulgaria | 8.78 (1.24; 38.1) | .015 | 7.58 (1.07; 33.2) | .015 |
| Croatia* |  | .993 |  | .993 |
| Cyprus* |  | .994 |  | .994 |
| Czech Republic | 1.51 (.30; 5.73) | .578 | 1.54 (.24; 5.84) | .578 |
| Denmark | 1.05 (.37; 2.67) | .842 | 1.10 (.38; 2.81) | .842 |
| Estonia | 0.71 (.16; 2.23) | .626 | 0.73 (.16; 2.30) | .626 |
| Finland* |  | .994 |  | .994 |
| France | 1.25 (.44; 3.17) | .703 | 1.20 (.42; 3.07) | .703 |
| Germany | 0.46 (.01; 1.66) | .322 | 0.47 (.01; 1.71) | .322 |
| Greece* |  | .994 |  | .994 |
| Hungary | 2.60 (.13; 16.9) | .443 | 2.39 (.12; 15.8) | .443 |
| Israel | 0.73 (.11; 2.68) | .572 | 0.65 (.10; 2.39) | .572 |
| Italy | 0.63 (.18; 1.77) | .408 | 0.62 (.17; 1.75) | .408 |
| Latvia* |  | .996 |  | .996 |
| Lithuania* |  | .993 |  | .993 |
| Luxembourg* |  | .984 |  | .984 |
| Malta | 2.76 (.41; 1.08) | .297 | 2.29 (.34; 9.09) | .297 |
| Netherlands | 0.38 (.01; 1.91) | .363 | 0.39 (.01; 1.98) | .364 |
| Poland | 4.73 (1.60; 12.4) | .003 | 4.58 (1.55; 12.0) | .003 |
| Portugal | 0.66 (.12; 2.87) | .629 | 0.69 (.11; 2.59) | .629 |
| Romania | 10.4 (3.63; 27.8) | <.001 | 9.82 (3.40; 26.3) | <.001 |
| Slovakia | 4.95 (.26; 29.7) | .169 | 4.55 (.23; 27.6) | .169 |
| Slovenia | 2.16 (.48; 7.02) | .270 | 2.09 (.46; 6.84) | .270 |
| Spain | 0.86 (.29; 2.19) | .586 | 0.76 (.26; 1.97) | .586 |
| Sweden | 1.15 (.43; 2.83) | .743 | 1.17 (.43; 2.87) | .743 |
| Switzerland | 0.79 (.25; 2.09) | .654 | 0.79 (.25; 2.09) | .654 |

***** No event of hospitalization for COVID-19 was reported in this country. As such, although these countries were kept in the model, reliable estimates could not be obtained and were therefore disregarded. Moreover, because the number of observations per country is low for most countries (only 13 countries have more than 100 observations), drawing conclusions based on these estimates was considered too speculative.

**Table S5.** Results based on the rare events logistic regression with a tau of 84/100,000

|  | Model 1  (without muscle strength) | | Model 2  (with muscle strength) | |  |
| --- | --- | --- | --- | --- | --- |
| **Variables** | OR (95% CI) | *p*-value | OR (95% CI) | *p*-value | |
| Intercept | 5.6E-4 (3.3E-4; 9.7E-4) | <.001 | 4.4E-4 (2.5E-4; 8.0E-4) | <.001 | |
| Age | 1.71(1.26; 2.31) | <.001 | 1.50 (1.10; 2.05) | .011 | |
| Sex (ref. Female) |  |  |  |  | |
| Male | .80 (.42; 1.50) | .482 | 1.30 (.64; 2.64) | .463 | |
| Height | 1.30 (.96; 1.76) | .086 | 1.47 (1.06; 2.03) | .021 | |
| Body mass index (ref. Normal) |  |  |  |  | |
| Overweight | 1.44 (.84; 2.47) | .182 | 1.54 (.90; 2.62) | .113 | |
| Obese | 2.00 (1.09; 3.68) | .026 | 2.10 (1.15; 3.85) | .016 | |
| Cardiovascular disease (ref. No) |  |  |  |  | |
| Yes | 1.05 (.65; 1.69) | .841 | 1.02 (.63; 1.63) | .946 | |
| Respiratory disease (ref. No) |  |  |  |  | |
| Yes | 1.63 (.77; 3.46) | .202 | 1.63 (.77; 3.45) | .205 | |
| Diabetes (ref. No) |  |  |  |  | |
| Yes | 1.23 (.63; 2.40) | .547 | 1.13 (.58; 2.18) | .724 | |
| Cancer (ref. No) |  |  |  |  | |
| Yes | .76 (.22; 2.59) | .656 | .70 (.20; 2.44) | .578 | |
| Rheumatoid arthritis (ref. No) |  |  |  |  | |
| Yes | 1.21 (.61; 2.38) | .591 | 1.11 (.56; 2.19) | .756 | |
| Chronic kidney disease (ref. No) |  |  |  |  | |
| Yes | 2.70 (.92; 7.97) | .072 | 2.65 (.91; 7.75) | .075 | |
| Muscle strength |  |  | .63 (.43; .92) | .016 | |

**Table S6.** Results in COVID-19 patients (N = 289)

|  | Model 1  (without muscle strength) | | Model 2  (with muscle strength) | |  |
| --- | --- | --- | --- | --- | --- |
| **Variables** | OR (95% CI) | *p*-value | OR (95% CI) | *p*-value | |
| Intercept | .15 (.07; .28) | <.001 | .11 (.05; .22) | <.001 | |
| Age | 1.72 (1.27; 2.36) | <.001 | 1.50 (1.08; 2.10) | .015 | |
| Sex (ref. Female) |  |  |  |  | |
| Male | .77 (.33; 1.76) | .537 | 1.55 (.54; 4.42) | .410 | |
| Height | 1.33 (.88; 2.04) | .181 | 1.48 (.96; 2.30) | .074 | |
| Body mass index (ref. Normal) |  |  |  |  | |
| Overweight | 1.80 (.88; 3.77) | .111 | 1.83 (.89; 3.86) | .103 | |
| Obese | 1.49 (.63; 3.50) | .357 | 1.50 (.63; 3.57) | .353 | |
| Cardiovascular disease (ref. No) |  |  |  |  | |
| Yes | 1.27 (.65; 2.52) | .479 | 1.14 (.57; 2.28) | .716 | |
| Respiratory disease (ref. No) |  |  |  |  | |
| Yes | 1.16 (.33; 3.49) | .805 | .97 (.28; 2.98) | .963 | |
| Diabetes (ref. No) |  |  |  |  | |
| Yes | .87 (.31; 2.21) | .782 | .73 (.25; 1.91) | .541 | |
| Cancer (ref. No) |  |  |  |  | |
| Yes | 1.02 (.14; 4.64) | .982 | .96 (.13; 4.41) | .964 | |
| Rheumatoid arthritis (ref. No) |  |  |  |  | |
| Yes | 1.10 (.40; 2.79) | .849 | .97 (.35; 2.49) | .950 | |
| Chronic kidney disease (ref. No) |  |  |  |  | |
| Yes | 1.35 (.25; 6.15) | .707 | .94 (.17; 4.45) | .936 | |
| Muscle strength |  |  | .56 (.33; .94) | .031 | |
